# Primary Intracranial Squamous Cell Carcinoma Arising from an Epidermoid Cyst: Successful Management with Subtotal Resection and Gamma Knife Radiosurgery in an Elderly Patient

**DOI:** 10.3390/curroncol33030158

**Published:** 2026-03-10

**Authors:** Won Gun Kwack, Hong Jun Kim

**Affiliations:** 1 Department of Pulmonary, Allergy, and Critical Care Medicine, College of Medicine, Kyung Hee University Hospital, 23 Kyung Hee Dae-ro, Dongdaemun-gu, Seoul 02447, Republic of Korea; wongunnim@naver.com; 2 Department of Medical Oncology, College of Medicine, Kyung Hee University Hospital, 23 Kyung Hee Dae-ro, Dongdaemun-gu, Seoul 02447, Republic of Korea

**Keywords:** intracranial squamous cell carcinoma, epidermoid cyst, malignant transformation, gamma knife radiosurgery, geriatric neuro-oncology

## Abstract

Primary intracranial squamous cell carcinoma (SCC) arising from an epidermoid cyst is an exceptionally rare and aggressive malignancy with a dismal prognosis. Conventional management typically involves gross total resection followed by wide-field radiotherapy; however, this intensive approach is often unfeasible for elderly or frail patients. We present a case of primary intracranial SCC in a 75-year-old woman who presented with rapid cochleovestibular deterioration. Imaging revealed subtle enlargement of a long-standing cerebellopontine angle epidermoid cyst. Subtotal resection was performed to preserve critical neurovascular structures adherent to the infiltrative tumor. Given the patient’s poor performance status and the risk of toxicity from broad-field radiation, adjuvant Gamma Knife radiosurgery (GKS) was selected as a focal salvage modality. Despite the limited surgical margin, the patient has maintained a progression-free status with no radiographic evidence of disease progression for 18 months without neurological decline. This case highlights the diagnostic challenge of malignant transformation disguised by radiologic mimicry and demonstrates that GKS can serve as an effective and tolerable adjuvant strategy. We propose that for high-risk patients precluded from intensive multimodal therapy, focused stereotactic irradiation offers a viable alternative to secure local control while preserving quality of life and systemic immune integrity.

## Simple Summary

Primary intracranial squamous cell carcinoma arising from a benign epidermoid cyst is an extremely rare and aggressive brain tumor. Conventional treatment involves extensive surgery and wide-field radiation, but this approach can be too dangerous for elderly or frail patients. We report the case of a 75-year-old woman with this diagnosis who could not undergo conventional aggressive therapy due to her poor health and the tumor’s proximity to critical brain structures. Instead, she received a partial removal of the tumor followed by Gamma Knife radiosurgery, a precise form of focused radiation. This tailored approach successfully controlled the cancer for 18 months without causing severe side effects. Our findings suggest that Gamma Knife radiosurgery can be an effective and safer alternative for high-risk patients who cannot tolerate standard intensive treatments.

## 1. Introduction

Intracranial epidermoid cysts are benign congenital lesions that constitute approximately 1% of all intracranial tumors. While these cysts typically follow an indolent clinical course, malignant transformation into squamous cell carcinoma (SCC) is an exceptionally rare phenomenon, with fewer than 100 cases reported in the literature to date [1,2]. Primary intracranial SCC arising from an epidermoid cyst is characterized by aggressive biological behavior, rapid progression, and a dismal prognosis, with a median survival often reported to be less than one year despite multimodal therapy [3]. The diagnosis is frequently delayed because the clinical and radiological presentation usually mimics that of benign lesions until rapid neurological deterioration occurs [4].

Due to the scarcity of cases, no consensus guidelines exist regarding the optimal management of this malignancy. The currently prevailing therapeutic strategy involves maximal surgical resection followed by adjuvant external beam radiotherapy (EBRT) or intensity-modulated radiotherapy (IMRT) to control microscopic residual disease [3,5]. However, applying this aggressive multimodal approach presents a significant challenge in real-world practice, particularly for elderly patients or those with significant comorbidities. As highlighted in recent oncologic literature, managing central nervous system (CNS) malignancies in geriatric populations requires a careful balance between achieving oncologic control and preserving quality of life, often necessitating modified treatment protocols when standard toxicities are prohibitive [6].

In such clinical scenarios where wide-field radiation is deemed unsafe, stereotactic radiosurgery (SRS), such as Gamma Knife surgery (GKS), could offer a viable alternative. By delivering high-dose radiation to a conformal target while sparing surrounding neurovascular structures, GKS may provide adequate local control with a more favorable safety profile. Although GKS is widely accepted for benign tumors or metastases, its role as a primary adjuvant modality for malignant epidermoid transformation remains understudied and is largely limited to palliative settings in existing reports [7].

Herein, we present a rare case of primary intracranial SCC transformed from a cerebellopontine angle (CPA) epidermoid cyst. Notably, the patient underwent subtotal resection followed by adjuvant GKS due to a poor general condition precluding conventional radiotherapy. Despite the limited surgical margin and omission of wide-field radiation, the patient has maintained radiographic disease stability without evidence of progression for 18 months. This case illustrates the potential utility of GKS as an effective and tolerable alternative adjuvant strategy for high-risk patients who are not candidates for intensive multimodal treatment.

## 2. Case Presentation

A 75-year-old woman presented to the neurosurgery outpatient clinic with a two-week history of rapidly progressive left-sided hearing loss, continuous tinnitus, and debilitating dizziness. Her past medical history was significant for hypertension and dyslipidemia, both managed with oral medications. Notably, she had a remote history of cerebral infarction occurring over a decade prior, for which she had been maintained on long-term antiplatelet therapy under regular neurologic surveillance. She denied any history of malignancy, head trauma, or prior neurosurgical interventions.

**Figure 1:**
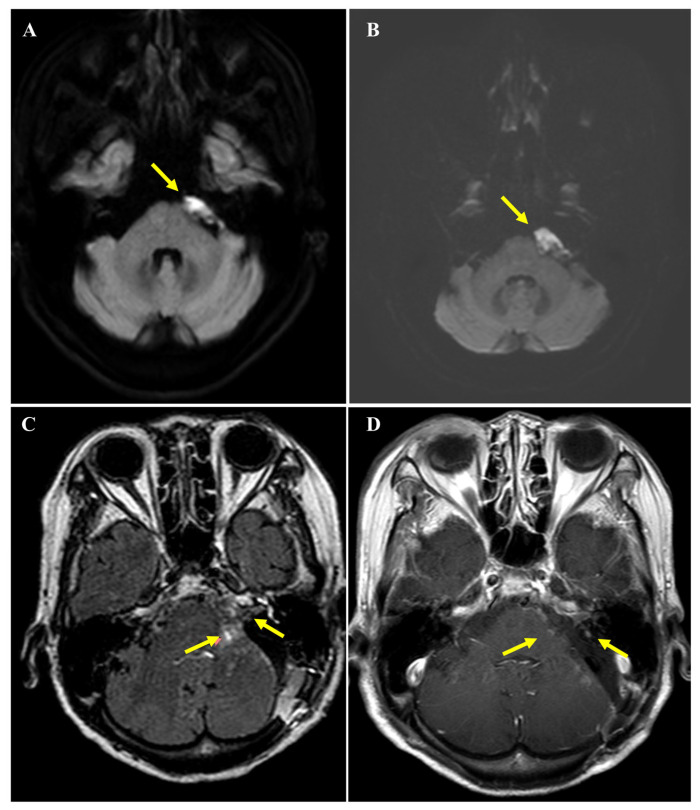
Serial magnetic resonance imaging (MRI) findings before surgery and prior to Gamma Knife radiosurgery. (**A**) Axial diffusion-weighted MRI demonstrating a well-defined lesion at the left cerebellopontine angle (arrow) with imaging features consistent with an intracranial epidermoid cyst, measuring approximately 2.3 × 1.1 cm, without significant mass effect on adjacent brainstem structures. (**B**) Follow-up axial diffusion-weighted MRI showing equivocal interval change with subtle enlargement of the left cerebellopontine angle lesion (arrow) to approximately 2.6 × 1.1 cm, while no definite mass effect or hydrocephalus is observed. (**C**) Axial post-contrast T2 fluid-attenuated inversion recovery MRI obtained on postoperative brain MRI before Gamma Knife radiosurgery demonstrating residual enhancing tumor components at the left cerebellopontine angle and extension into the internal auditory canal (arrows) following subtotal resection. Mild residual mass effect on the left pons and slight displacement of the ipsilateral facial and vestibulocochlear nerve complex are also noted. (**D**) Axial contrast-enhanced T1-weighted spin-echo MRI obtained 18 months after Gamma Knife radiosurgery demonstrating marked reduction of the residual tumor at the left cerebellopontine angle and decreased extent of enhancing lesions within the left internal auditory canal (arrows), consistent with radiographic response and no evidence of disease progression.

Routine surveillance brain magnetic resonance imaging (MRI) performed three years prior to the current presentation had identified a 2.3 × 1.1 cm non-enhancing lesion in the left CPA. The lesion demonstrated restricted diffusion characteristic of an intracranial epidermoid cyst and had shown no interval change compared to imaging studies from the preceding decade (Figure 1A). However, approximately two weeks before presentation, the patient experienced a sudden exacerbation of dizziness that severely compromised her ability to ambulate independently. An urgent MRI showed no significant mass effect or hydrocephalus, and her symptoms transiently responded to conservative medical management. Unfortunately, this improvement was short-lived; within days, she developed persistent, high-pitched tinnitus and subjective profound hearing loss on the left side. A follow-up diffusion-weighted MRI revealed equivocal morphological changes with a subtle enlargement of the lesion to 2.6 × 1.1 cm (Figure 1B). Although the size increase was minimal, the acute onset of cochleovestibular symptoms raised concern for clinically significant disease progression, ultimately leading to a decision to proceed with surgical intervention.

**Figure 2:**
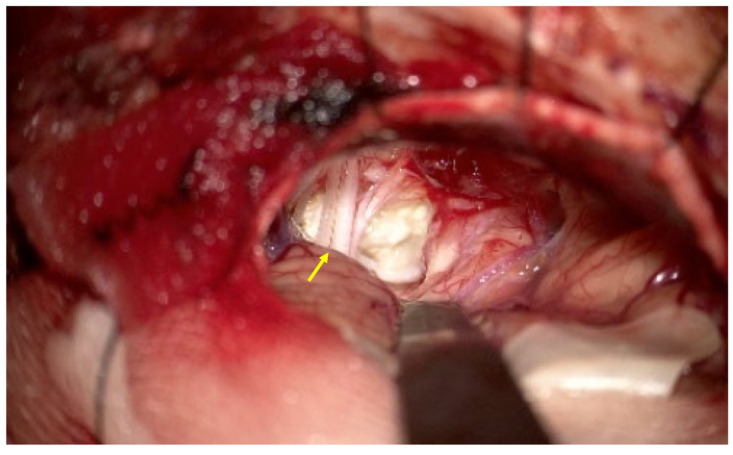
Intraoperative view of the left cerebellopontine angle lesion obtained via a retrosigmoid approach. The exposed tumor demonstrates the characteristic pearly white, flaky appearance consistent with an epidermoid cyst component. Adjacent cranial nerve bundles are visibly displaced and stretched over the tumor surface (arrow), reflecting the local mass effect within the cerebellopontine angle cistern. Although the superficial component showed benign macroscopic features, the deeper portion exhibited firm adhesion to the brainstem, corresponding to the malignant component identified on histopathologic examination.

The patient was admitted for elective surgery several weeks after the initial outpatient evaluation. On preoperative examination, she was alert and oriented but exhibited left-sided facial hypesthesia and severe sensorineural hearing loss. Prior to surgical intervention, a comprehensive systemic evaluation was performed to exclude metastatic disease. Whole-body imaging, including 18F-FDG positron emission tomography/computed tomography, revealed no evidence of a primary extracranial malignancy, and otolaryngologic examination, including endoscopic evaluation of the upper aerodigestive tract, revealed no suspicious lesions. A left retrosigmoid suboccipital craniotomy was performed with image-guided navigation. Upon opening the dura, the surgical field revealed marked displacement of the sixth, seventh, eighth, and lower cranial nerves (Figure 2). The lesion consisted of two distinct components. The first was a typical epidermoid cyst—a soft, pearly white, avascular mass—which was easily debulked via suction. Beneath this layer, however, a firm, vascularized solid mass was identified, extending into the internal auditory canal (IAC) and adhering tightly to the brainstem and vestibulocochlear nerve complex. This solid component exhibited frequent contact bleeding and lacked a clear dissection plane, initially mimicking a hemorrhagic vestibular schwannoma. Intraoperative frozen-section analysis confirmed the presence of malignant cells with squamous differentiation. Given the tumor’s firm adherence to critical neurovascular structures and the high risk of permanent neurological deficit, a decision was made to perform a maximal safe subtotal resection rather than risking gross total removal.

**Figure 3:**
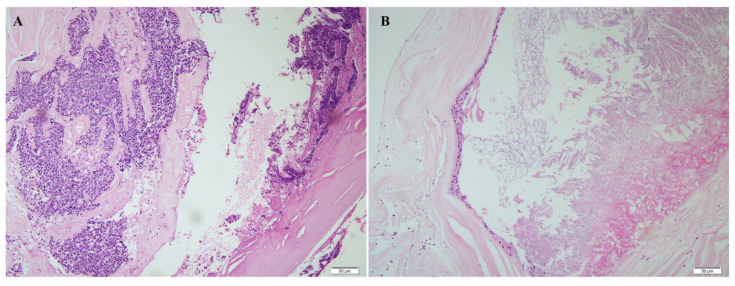
Histopathologic features of malignant transformation arising from an intracranial epidermoid cyst. (**A**) Hematoxylin and eosin (H&E)-stained section demonstrating invasive squamous cell carcinoma with sheets of atypical squamous cells, nuclear pleomorphism, and keratinization infiltrating the surrounding tissue. Scale bar = 50 μm. (**B**) Adjacent area showing typical features of a benign epidermoid cyst, characterized by laminated keratin debris lined by stratified squamous epithelium without cytologic atypia. Scale bar = 50 μm. The images represent routine H&E staining without additional color annotation or artificial color enhancement.

Final histopathologic examination of the resected specimen demonstrated invasive SCC (Figure 3A) arising within the background of a benign epidermoid cyst (Figure 3B), confirming the diagnosis of primary intracranial SCC. To guide potential adjuvant therapies, next-generation sequencing (NGS) was performed using a targeted solid tumor panel. The analysis identified pathogenic somatic mutations in TP53, KMT2D, ZFHX3, and RNF43. Additionally, copy number variation analysis revealed FGFR1 amplification and multiple chromosomal gains. These molecular findings, particularly the TP53 mutation and FGFR1 amplification, suggested an aggressive tumor biology and potential therapeutic implications.

Postoperatively, the patient’s case was reviewed by a multidisciplinary tumor board. While definitive EBRT or IMRT could be considered as a viable option of care for malignant CPA tumors, these options were deemed suboptimal for this patient. Her advanced age, limited functional reserve, and history of cerebrovascular disease raised concerns regarding her ability to tolerate a prolonged 6-week course of wide-field radiation. Furthermore, the residual tumor’s proximity to the brainstem posed a high risk of radiation-induced toxicity. Consequently, GKS was selected as a focused, hypofractionated salvage modality. Pre-radiosurgical MRI visualized residual enhancing nodules in the CPA and IAC (Figure 1C). SRS was delivered to these two target volumes (352 mm^3^ and 219 mm^3^) with a marginal dose of 18 Gy at the 60% isodose line (maximum dose: 30 Gy). The procedure was completed without acute complications. At 18 months post-radiosurgery, serial follow-up MRIs have demonstrated stable disease with no evidence of local recurrence or distant metastasis (Figure 1D), and the patient remains neurologically stable at her baseline.

## 3. Discussion

This case highlights several clinically meaningful aspects in the management of primary intracranial SCC arising from an epidermoid cyst. Our patient demonstrated rapid neurological deterioration despite minimal radiologic interval change, emphasizing the diagnostic challenge associated with malignant transformation in long-standing benign-appearing lesions. Intraoperatively, the coexistence of typical epidermoid cyst material and an infiltrative solid tumor component further underscored the heterogeneous biological behavior of this rare entity [1,3,8]. Importantly, although gross total resection and adjuvant wide-field radiotherapy are advocated [3,5], such an approach was not feasible because of the patient’s limited functional reserve and the proximity of residual disease to critical neurovascular structures. The favorable local control achieved with subtotal resection followed by GKS suggests that focused stereotactic irradiation may represent a viable alternative strategy in carefully selected high-risk patients. This outcome adds to the limited existing evidence regarding radiosurgical management in malignant epidermoid transformation and supports consideration of individualized treatment adaptation when intensive multimodal therapy is not tolerated.

The rarity of malignant transformation in intracranial epidermoid cysts, with an incidence estimated at less than 0.005% of brain tumors, poses a significant challenge for timely diagnosis and standardized management [1,9]. Radiologically, the malignant component often retains the benign features of the precursor cyst, leading to a so-called radiologic mimicry phenomenon where significant tumor progression is masked by a stable cystic appearance on conventional MRI [4]. As observed in our case, the discrepancy between minimal imaging changes and rapid clinical deterioration—characterized by new-onset cranial nerve deficits or intractable pain—serves as a critical red flag necessitating immediate intervention, regardless of radiologic stability [9]. Furthermore, while the current paradigm of gross total resection followed by EBRT offers the best theoretical chance for cure, its real-world application is frequently limited. Achieving gross total resection is often precluded by the tumor’s tenacious adherence to the brainstem and cranial nerves, as reported in up to 40% of cases due to the risk of devastating neurological morbidity [3,10]. Consequently, in the setting of inevitable residual disease, the reliance on wide-field EBRT becomes a double-edged sword, offering locoregional control at the cost of potential neurotoxicity, particularly in elderly or frail populations where physiological reserve is diminished [11].

In light of the surgical limitations imposed by neurovascular adherence, the selection of adjuvant radiotherapy becomes pivotal for disease control. While conventional fractionated radiotherapy or IMRT provides broad coverage, the anatomical constraints of the CPA present unique challenges. The residual tumor in our patient was intimately draped over the brainstem and vestibulocochlear nerve complex, areas where the collateral damage from wide-field radiation can lead to an increased risk of sensorineural hearing loss, facial neuropathy, or brainstem toxicity [6]. For elderly patients with limited physiological reserve, the burden of a prolonged 6-week treatment course further compromises compliance and overall quality of life. In this context, GKS offers a distinct dosimetric advantage by creating a steep dose fall-off gradient that precisely conforms to the irregular tumor geometry while sparing adjacent critical structures. This precision reflects the advances in functional stereotaxy, which enables the delivery of ablative doses while prioritizing the preservation of neurological function in complex anatomical locations [12]. Emerging evidence regarding intracranial malignancies in geriatric populations suggests that hypofractionated stereotactic approaches can achieve comparable local control to whole-brain or wide-field radiation but with significantly reduced neurocognitive toxicity and preservation of functional independence [7]. Furthermore, recent studies indicate that avoiding large treatment volumes minimizes radiation-induced lymphopenia, a factor increasingly recognized to correlate with poorer survival outcomes in brain tumor patients [13]. By adopting a highly focused radiosurgical strategy, we aimed to spare the circulating lymphocyte pool and preserve the patient’s systemic immune integrity, which is particularly crucial in the elderly. Thus, we were able to deliver a biologically effective focal dose to the infiltrative component without precipitating the rapid neurological decline often associated with aggressive wide-field irradiation in frail subjects.

From both a physical and radiobiological standpoint, Gamma Knife stereotactic radiosurgery offers several advantages over conventional IMRT-based external beam radiotherapy. The cobalt-60-based fixed collimator system produces minimal transmission leakage and a sharper penumbra compared with multi-leaf collimators, enabling highly conformal dose distributions with steep dose fall-off—an important consideration when treating lesions adjacent to the brainstem and cranial nerves [12]. In addition, the rigid stereotactic head frame ensures submillimetric targeting accuracy, further reducing unintended dose to surrounding critical structures. Radiobiologically, high-dose single-fraction irradiation may induce enhanced tumor cell kill through endothelial damage and vascular collapse, while also promoting immunogenic tumor cell death and potential immune-mediated effects, which have been increasingly recognized as contributing to durable local control following stereotactic radiosurgery [13,14].

Given the aggressive nature of the tumor, we performed NGS to explore potential systemic therapeutic options, identifying pathogenic mutations in TP53 and FGFR1 amplification. While TP53 alterations are frequently associated with treatment resistance and poor prognosis in SCC [15], the clinical utility of targeted therapy for primary intracranial SCC remains severely limited. Currently, there is no established standard chemotherapy regimen for this rare entity, and the efficacy of systemic agents is further compromised by the blood–brain barrier, which restricts the delivery of cytotoxic drugs to the CNS sanctuary site [16]. Although immunotherapy has shown promise in extracranial SCC, its application in intracranial cases is largely theoretical, with a lack of robust clinical evidence to support its routine use [17]. Consequently, in the absence of effective systemic salvage options, durable disease control relies almost exclusively on aggressive local management. The identification of high-risk molecular features in our patient paradoxically reinforced the decision to pursue maximal local control via radiosurgery rather than relying on unproven systemic therapies. This underscores that for primary intracranial SCC, optimizing the synergy between surgery and precise adjuvant radiation remains a key determinant of clinical outcome.

One important limitation of this case should be acknowledged. Although histopathologic examination demonstrated invasive squamous cell carcinoma arising in the background of a benign epidermoid cyst, a definitive microscopic transition zone between the benign squamous epithelium and malignant components could not be clearly identified. Demonstration of such a transition zone is considered an important criterion to unequivocally confirm malignant transformation rather than metastatic disease. In the present case, this limitation may be attributable to sampling constraints inherent to subtotal resection and the heterogeneous architecture of the lesion. Nevertheless, the diagnosis of primary intracranial squamous cell carcinoma was supported by the coexistence of benign and malignant components within the same lesion, characteristic intraoperative findings, and a comprehensive systemic evaluation excluding an extracranial primary source.

Based on our experience, we propose a tailored therapeutic framework where GKS is considered not merely as a salvage option for recurrence, but as a primary adjuvant alternative for a specific subset of high-risk patients. Candidates for this approach should include elderly or frail individuals with significant comorbidities, those with residual disease intimately adherent to the brainstem, and patients for whom the toxicity of wide-field radiation is prohibitive. In this context, radiosurgery does not seek to replace maximal resection or standard radiotherapy but rather complements subtotal resection by securing local control while preserving functional integrity [14]. This strategy aligns with the growing emphasis in geriatric neuro-oncology on balancing oncologic outcomes with quality of life [18]. Moving forward, given the extreme rarity of this malignancy, the establishment of multi-institutional registries is essential to validate this approach and accumulate robust data. Future research should also integrate molecular profiling, as seen in our case, to identify potential biomarkers that might stratify patients who would benefit most from such focused, biology-driven local therapies. Until then, individualized treatment adaptation remains the cornerstone of management for these challenging cases.

## 4. Conclusions

This case demonstrates that primary intracranial SCC arising from an epidermoid cyst can be successfully controlled using a tailored multimodal strategy in complex clinical scenarios where conventional treatment approaches are difficult to apply. In an elderly patient with limited physiological reserve and residual disease located adjacent to critical neurovascular structures, subtotal resection followed by adjuvant GKS appeared to provide durable local control without significant treatment-related morbidity. Our findings highlight the importance of maintaining clinical suspicion despite subtle radiologic changes and emphasize the value of individualized treatment adaptation in rare CNS malignancies. Although wide-field radiotherapy remains commonly adopted in reported cases, SRS may represent a valuable alternative for carefully selected high-risk patients. Further accumulation of multicenter data and incorporation of molecular profiling will be essential to refine therapeutic strategies and improve outcomes for this rare clinical entity. Given the extreme rarity of this entity, the development of multi-institutional rare tumor registries will be essential to enable systematic data collection, facilitate collaborative research, and ultimately refine evidence-based management strategies for primary intracranial squamous cell carcinoma.

## Data Availability

The data supporting the findings of this study are available within the article. No new datasets were generated or analyzed beyond the clinical information presented. Additional data are not publicly available due to patient privacy and ethical restrictions.

## Abbreviations

The following abbreviations are used in this manuscript:

SCC	Squamous cell carcinoma
EBRT	External beam radiotherapy
IMRT	Intensity-modulated radiotherapy
CNS	Central nervous system
SRS	Stereotactic radiosurgery
GKS	Gamma Knife surgery
CPA	Cerebellopontine angle
MRI	Magnetic resonance imaging
IAC	Internal auditory canal
NGS	Next-generation sequencing
